# The Sulfotransferase *SULT1C2* Is Epigenetically Activated and Transcriptionally Induced by Tobacco Exposure and Is Associated with Patient Outcome in Lung Adenocarcinoma

**DOI:** 10.3390/ijerph19010416

**Published:** 2021-12-31

**Authors:** Candace Johnson, Daniel J. Mullen, Suhaida A. Selamat, Mihaela Campan, Ite A. Offringa, Crystal N. Marconett

**Affiliations:** 1 Department of Surgery, Keck School of Medicine, University of Southern California, Los Angeles, CA 90089-9520, USA; fudgecandi@yahoo.com (C.J.); dmullen@usc.edu (D.J.M.); Suhaida.selamat@merck.com (S.A.S.); velicesc@usc.edu (M.C.); ilaird@usc.edu (I.A.O.); 2 Department of Biochemistry and Molecular Medicine, Keck School of Medicine, University of Southern California, Los Angeles, CA 90089-9520, USA; 3 Norris Comprehensive Cancer Center, Keck School of Medicine, University of Southern California, Los Angeles, CA 90089-9520, USA

**Keywords:** cigarette smoke condensate, racial disparities, lung cancer, epigenetic/epigenomic influence on cancer

## Abstract

Lung cancer is the leading cause of cancer-related death. Tobacco exposure is associated with 80–90% of lung cancer cases. The *SULT1C2* sulfotransferase modifies xenobiotic compounds to enhance secretion but can also render these compounds carcinogenic. To determine if *SULT1C2* contributes to tobacco-related carcinogenesis in the lung, we analyzed the expression and epigenetic state of *SULT1C2* in human lung adenocarcinoma (LUAD) samples and in LUAD cell lines exposed to cigarette smoke condensate (CSC). *SULT1C2* expression was significantly positively correlated to overall LUAD patient survival in smokers, was elevated in LUAD tumors compared to adjacent non-tumor lung, and was significantly correlated with levels of patient exposure to tobacco smoke. *SULT1C2* promoter DNA methylation was inversely correlated with expression in LUAD, and hypomethylation of the *SULT1C2* promoter was observed in Asian patients, as compared to Caucasians. In vitro analysis of LUAD cell lines indicates that CSC stimulates expression of *SULT1C2* in a dose-dependent and cell-line-specific manner. In vitro methylation of the *SULT1C2* promoter significantly decreased transcriptional activity of a reporter plasmid, and *SULT1C2* expression was activated by the DNA demethylating agent 5-Aza-2′-deoxycytidine in a cell line in which the *SULT1C2* promoter was hypermethylated. An aryl hydrocarbon receptor (AHR) binding site was detected spanning critical methylation sites upstream of *SULT1C2*. CSC exposure significantly increased AHR binding to this predicted binding site in the *SULT1C2* promoter in multiple lung cell lines. Our data suggest that CSC exposure leads to activation of the AHR transcription factor, increased binding to the *SULT1C2* promoter, and upregulation of *SULT1C2* expression and that this process is inhibited by DNA methylation at the *SULT1C2* locus. Additionally, our results suggest that the level of *SULT1C2* promoter methylation and gene expression in normal lung varies depending on the race of the patient, which could in part reflect the molecular mechanisms of racial disparities seen in lung cellular responses to cigarette smoke exposure.

## 1. Background

Lung cancer is the most frequent cause of cancer-related death worldwide [1] and is the leading cause of cancer deaths of both men and women in the United States [2]. Lung adenocarcinoma (LUAD) is the most common histological subtype of lung cancer and the predominant form found among Asian and never-smoker patients [3,4]. In North America, 90% of the men and 75% of the women with lung cancer are current or former smokers. However, in Taiwan, only 7% of the female patients with lung adenocarcinoma are smokers [5]. Several studies have carefully investigated possible mechanisms that account for differences in LUAD occurrence between Asian and Caucasian patients [6,7].

Depending on social, cultural, and genetic differences, exposure to environmental toxins, including environmental tobacco smoke (ETS), varies among different racial groups [8,9]. Complex enzymatic systems have evolved to solubilize and secrete harmful toxins such as those found in cigarette smoke; however, it is largely unknown how these vary across racial groups. Sulfotransferase Family 1C member 2 (*SULT1C2*) is a critical component of the environmental detoxification pathway—a sulfotransferase that transfers a sulfur group to various substrates, e.g., xenobiotics such as drugs and chemical carcinogens [10]. Of the eleven sulfotransferase (SULT) family members, *SULT1C2* showed the strongest enzymatic activity toward cigarette smoke and was the only SULT family member that did not affect endogenous chemicals, such as estrogen [11]. In most cases, sulfonation of xenobiotics and small endogenous substrates detoxifies the body by increasing water solubility so the compound is cleared via urine or bile. However, in the case of certain environmental toxins such as chemicals present in cigarette smoke, SULTs can metabolically activate substrates into electrophiles that can be both carcinogenic and mutagenic [12,13]. It has been established that *SULT1C2* is expressed in human stomach, kidney, and fetal liver [14], and recently, expression was also observed in the colorectal adenocarcinoma cell line LS180 [15]. Smoking-associated cancers occur in all of these tissues [16,17]. However, the effect of cigarette smoke exposure on *SULT1C2* in lung is unknown [13].

Xenobiotic exposure can induce expression of detoxifying enzymes through activation of the aryl hydrocarbon receptor (AHR). Chemicals found in cigarette smoke are known ligands of AHR, which, when liganded, translocates to the nucleus where it partners with aryl hydrocarbon receptor nuclear translocator (ARNT) and acts as a transcription factor to upregulate expression of phase I detoxifying enzymes such as *CYP1B1* [18] as well as phase II enzymes [19,20,21,22]. The AHR recognition sequence, also known as the xenobiotic response element (XRE), is 5′-G/T.N.G.C.G.T.G.A/C.G/C.A-3′, which contains a cytosine followed by guanine (CpG) dinucleotide demonstrated to disrupt AHR binding in certain biological contexts [23,24]. Transcriptional silencing by methylation of deoxyribonucleic acid (DNA) at CpG dinucleotides is found at or near promoters or enhancers clustered in regions dubbed “CpG islands” [25]. However, 45% of tissue-specific promoters are nearly devoid of CpGs [26,27]. Controversy exists about the role non-CpG island DNA methylation events play in gene regulation. Specific genes with CpG-poor promoters are expressed when methylated [28,29], while others show an inverse correlation between DNA methylation and gene expression similar to that observed with CpG island promoters [30,31]. *SULT1C2* has a non-CpG island promoter that is normally methylated and silenced in adult lung tissue; however, the role of DNA methylation in regulation of the CpG-island poor promoter of *SULT1C2* when exposed to environmental chemicals such as cigarette smoke has not been previously characterized. We therefore hypothesized that DNA methylation of the *SULT1C2* promoter may control *SULT1C2* expression, leading to altered metabolism of xenobiotic chemicals such as cigarette smoke, and that this regulation may vary by race. To test this, we evaluated the expression and regulation of *SULT1C2* in LUAD and non-tumor lung samples from different population groups, in LUAD cell lines, and under CSC exposure conditions, to determine if epigenetic regulation of *SULT1C2* could be contributing to the previously observed racial disparities in lung adenocarcinoma incidence.

## 2. Methods

### 2.1. Reagents and Antibodies

Culture grade DMSO and rabbit polyclonal antibody for *SULT1C2* (SC-130274) were purchased from Santa Cruz Biotechnology (Santa Cruz, CA, USA). The actin antibody (AAN01) was purchased from Cytoskeleton Inc. (Denver, CO, USA). Cigarette smoke condensate (CSC; #NC9028647) was purchased from Murty Pharmaceuticals Inc. (Lexington, KY, USA). Trypsin-EDTA was obtained from USC Cell Culture Core Facility (Los Angeles, CA, USA).

### 2.2. Cell Culture

Bronchial epithelial cell line BEAS-2B (ATCC#CRL9609) was obtained from American Type Culture Collection (Manassas, VA, USA). PC3 (herein called PC3_LUAD) lung adenocarcinoma cell line (JCRB0077) was obtained from the Japanese Cancer Research Resources Bank (Osaka, Japan) and has no relation to the ubiquitous prostate cancer cell line PC3; they were derived from different donors. H2347 lung adenocarcinoma cell line was a kind gift from Dr. Eric Haura. Cancer cell lines were maintained in RPMI-1640 from Mediatech (10-040-CV, Manassas, VA, USA), and the BEAS-2B cell line was maintained in modified Eagle’s medium (USC Cell Culture Core Facility). All media were supplemented with 10% fetal bovine serum, Genesee Scientific (25-514, El Cajon, CA, USA), and 100U penicillin/streptomycin and grown in a humidified chamber with 5% CO_2_ at 37 °C. CSC (40 mg/mL) was diluted in DMSO from Corning (Manassas, VA, USA) and diluted 1:1000 in media prior to cell exposure. DMSO was used as the vehicle control for all experiments.

### 2.3. RNA Isolation and Quantitative Real-Time PCR (qRT-PCR)

Total RNA from BEAS-2B, PC3_LUAD, and H2347 cells treated with CSC or 5-Aza-CdR was isolated with Qiagen AllPrep DNA/RNA/Protein Kit (Valencia, CA, USA) according to manufacturer’s protocol. RNA was quantified using the Implen NanoPhotometer Pearl (Westlake Village, CA, USA). Total RNA (500 ng) was converted to cDNA using iScript cDNA Synthesis Kit (Hercules, CA, USA). The cDNA reaction product was amplified with primers listed in Table 1. PCR products were analyzed using the Bio-Rad Real-Time System (Hercules, CA, USA) measuring Sybr Green (Bio-Rad).

### 2.4. Next-Generation Sequencing Analysis

RNAseq from BEAS-2B (sh-Control rep1) was downloaded from Gene Expression Omnibus (GEO) record GSE55215 [32]. RNAseq from PC3_LUAD and H2347 was downloaded from GEO record GSE110024 [33]. FASTQ files were cleaned using fastp [32] to retain only those reads for which >90% of the reads have a quality score >30. Reads were trimmed to remove degenerate sequence at the 5′ and 3′ ends, and the resulting cleaned reads underwent alignment to the hg19 genome and featureCount quantitation using RNA STAR [34]. BAM alignments were visualized using the Integrated Genomics Viewer (IGVv2.8.2) [35]. For methylation analysis, Control_24h_1, Control_24h_2, and Control_24h_3 files of BEAS-2B reduced-representation bisulfite sequencing (RRBS) were downloaded from GEO record GSE155615 [36]. Control (untreated) sample files were merged and then aligned to the masked hg19 genome using bwameth [37]. Shotgun whole-genome bisulfite sequencing (sWGBS) was downloaded from the Database of Transcription Start Sites (DBTSS) (https://dbtss.hgc.jp/ accessed on 15 July 2021) [38] and aligned to the masked hg19 genome using BSMAP [39]. BAM-level alignment files were visualized using Integrated Genomics Viewer (IGV) in bisulfite mode [35].

### 2.5. The Cancer Genome Atlas (TCGA) Datasets

TCGA gene expression data used in this study were given in the form of log2-transformed fragments per kilobase of transcript per million mapped reads, upper quartile normalized (FPKM-UQ) values, which were mapped to the hg38 human genome and annotated to GENCODE v22 genes. DNA methylation β-values were given for HM450 probes. For this study, two different TCGA LUAD gene expression and DNA methylation datasets were used. The first contained samples with unmatched gene expression and DNA methylation and was used for analyses where only gene expression or DNA methylation was analyzed. The second dataset contained only samples with matched gene expression and DNA methylation and was used when gene expression and DNA methylation values were compared together. Duplicate samples from the same patient were removed (leaving 1 per patient), as well as samples lacking race, age, gender, and smoking history. A total of 53 adjacent non-tumor and 429 LUAD tumor gene expression samples, and 26 adjacent non-tumor and 390 LUAD tumor DNA methylation samples were included in the first dataset, while 16 adjacent non-tumor and 386 LUAD tumor samples with both gene expression and DNA methylation data were included in the second dataset. Expression correlations between *AHR* and either *SULT1C2* or *CYB1B1* were generated using data preprocessed in TIMER2.0 [40], which was sourced from expression data generated by TCGA and processed according to their published algorithms [41].

### 2.6. Survival Curves

Overall survival curves for expression of *SULT1C2* were generated using KMplot [42] on lung cancer, split by upper quartile expression and subsequently split by smoking status (ever-smokers *n* = 820; never-smokers *n* = 205). Overall survival curves for methylation at cg13968390 were generated using TCGA data obtained from TCGAbiolinks [43] for LUAD split by upper and lower quartile.

### 2.7. Microarray Analysis

Expression data generated by the Early Detection Research Network (EDRN) were originally run on the Illumina Sentrix-6 whole-genome expression bead chip (WG-6) microarray platform. Preprocessed and normalized data were downloaded from GEO (GSE32867) and used for all subsequent analyses. Methylation data generated on the Illumina Infinium 27K array by the EDRN and on previously published samples from the Ontario Tumor Bank (OTB) were downloaded from GEO (GSE32861 and GSE32866, respectively) [44]. Beta-values were calculated by dividing the methylation value at a given CpG by the sum of both methylated (M) and unmethylated signal (U) for that probe [M/(U + M)] [45].

### 2.8. SULT1C2 Promoter Construction

The *SULT1C2* promoter from −1271 from the transcriptional start site (TSS) through +535 in the 5′ untranslated region (UTR) was PCR-amplified from genomic DNA from lung cancer cells using Phusion High-Fidelity DNA Polymerase from New England Biolabs (Ipswich, MA, USA) using primers listed in Table 1. The gel fragment was purified using Qiaquick Gel Extraction kit from Qiagen (Valencia, CA, USA). The fragment was then inserted into the CpG-less vector using Instant Sticky-end Ligase Master Mix from New England Biolabs (NEB) (Ipswich, MA, USA). The plasmid was transformed into Invitrogen *E. coli* PIR1cells (Grand Island, NY, USA). The promoter sequence was verified using Genewiz (La Jolla, CA, USA).

### 2.9. In Vitro Methylation

The CpG-less vector containing the *SULT1C2* promoter (pCpGL-*SULT1C2*) was incubated with *Sss*I (2.5 U/μg) in the presence of 160 uM S-Adenosylmethionine (SAM), both from New England Biolabs (Ipswich, MA, USA) overnight. This was repeated for a second overnight treatment. Methylation of plasmids was confirmed by digestion of methylation-sensitive restriction enzymes *Hpa*II and *Hha*I, and methylation-insensitive *Msp*I from New England Biolabs (Ipswich, MA, USA).

### 2.10. Transfection and Luciferase Assay

PC3_LUAD cells were transfected with 800 ng of methylated and unmethylated pCpGL-*SULT1C2* luciferase reporter vector and 200 ng of Renilla luciferase using Invitrogen Lipofectamine 2000 in low-serum Optimem media (Grand Island, NY, USA). H2347 and BEAS-2B cells were transfected using FuGene HD transfection reagent from Promega (Madison, WI, USA) using the same vector amounts. Twenty-four hours post-transfection, medium was removed and replaced with medium containing 10% fetal bovine serum (FBS), 20 μg/mL of CSC or dimethylsufoxide (DMSO) vehicle control in the absence of antibiotics for 24 h. Cells were washed with PBS and lysed with Promega passive lysis buffer (Madison, WI, USA). Cell lysates were freeze/thawed and assayed for firefly and renilla luciferase activity using Promega Dual-Luciferase Reporter Assay System on a Promega Glomax Luminometer. pCpGL and constitutive cytomegalovirus (CMV) vectors were used for negative and positive controls, respectively. Experiments were performed as technical triplicates in three independent experiments.

### 2.11. Aza-CdR Treatment

BEAS-2B, PC3_LUAD, and H2347 cell lines were plated 24 h prior to treatment. Cells were treated with the indicated concentrations of 5-Aza-CdR from Sigma Chemical Co. (St Louis, MO, USA) for 24 h. Cells were allowed to recover and replicate for 72 h post drug removal. On the third day, cells were washed with cold PBS and harvested for RNA and DNA.

### 2.12. MethyLight Assay

One microgram of DNA was bisulfite-treated using the EZ DNA Methylation kit from Zymo Research (D5002, Irvine, CA, USA). Bisulfite-treated DNA was probed with MethyLight primers and probe as described in Table 1. Control and treated DNA was incubated with Taq Man enzyme from Applied BioSystems (Carlsbad, CA, USA), primers, and probe in 30 µL reactions and analyzed with Alu repeats used to normalize input DNA as previously published [46].

### 2.13. Chromatin Immunoprecipitation (ChIP) Assay

PC3_LUAD, H2347, and BEAS-2B cells were grown to 85% confluency in 150 mm plates and were treated with DMSO or 20 μg/mL CSC for the indicated times. Cells were cross-linked using 1% formaldehyde for 10 min followed by quenching with 125 mM Glycine (Sigma). Cells were lysed in 800 μL of cell lysis buffer with protease inhibitors from Sigma-Aldrich (#P2714, St. Louis, MO, USA) and incubated on ice for 20 min, then DNA was sonicated to lengths between 200 bp and 1000 bp. Input was 1% of the total lysate. Chromatin-immunoprecipitation (ChIP)-grade AHR antibody (ab84833) from Abcam (Cambridge, MA, USA) and control IgG (sc-66931) from Santa Cruz Biotechnology were used to precipitate DNA. Purified ChIP DNA was then purified by phenol-chloroform extraction and quantified using Implen Nanophotometer Pearl (Westlake Village, CA, USA).

### 2.14. Statistical Analysis

Multivariate analysis of TCGA data was performed using sample type, reported gender, age, smoking history, and race as covariates. *p* values for significance of comparisons between two paired variables were obtained using a two-sided paired *t* test; the only exception was DNA methylation beta-value comparisons in the OTB database, where we had a hypothesis for the direction of change prior to performing the test. In that instance, a one-sided paired *t* test was performed. For TCGA data, unpaired *t* tests were used because the AdjNTL and LUAD tumor samples were derived from different individuals. *p* values for significance determination between three or more variables were performed using ANOVA. Q values were used for multiple comparisons correction on the effect of race on *SULT1C2* promoter methylation. The *p* and Q values for significance threshold were set at *p*/Q < 0.05. Throughout this study, * is used to indicate significance values < 0.05, ** indicates < 0.01, and *** indicates < 0.001.

## 3. Results

Expression of *SULT1C2* is correlated with overall LUAD patient survival and cigarette smoke exposure, and cigarette smoke condensate can induce *SULT1C2* in lung cell lines.

**Figure 1:**
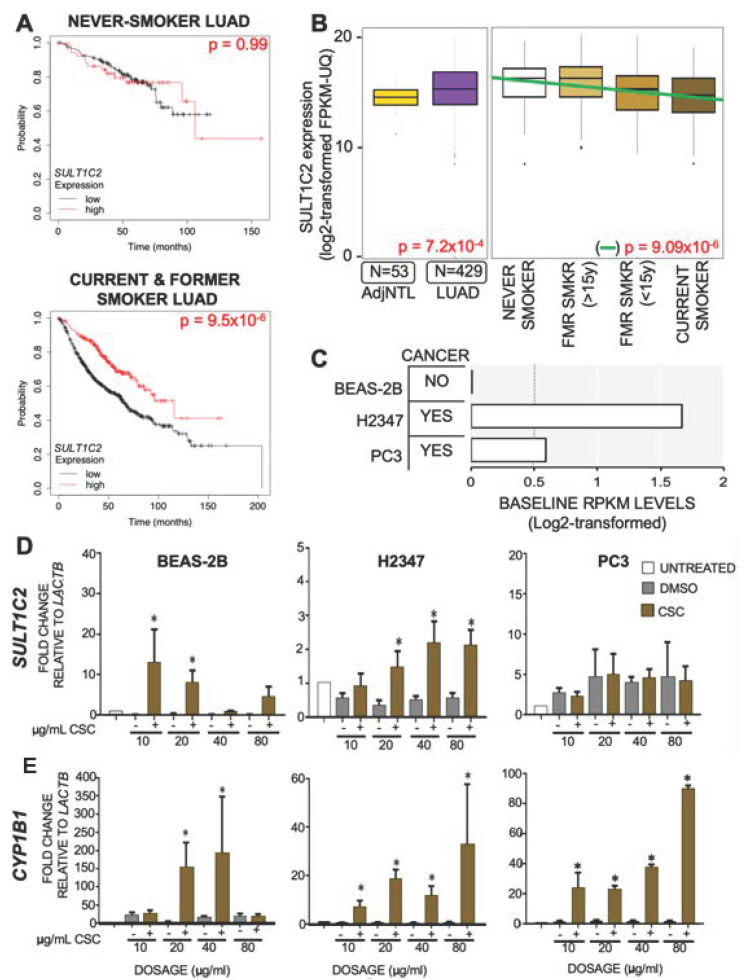
Expression of *SULT1C2* affects overall LUAD patient survival and is activated by cigarette smoke. (**A**) Survival plots of LUAD patients stratified by *SULT1C2* expression. Upper panel includes patients that never smoked; lower panel includes only patients with documented smoke exposure. KMplot [47] was used to stratify samples by upper quartile. (**B**) Left: distribution of *SULT1C2* in human LUAD patient tumors and adjacent non-tumor lung from TCGA. Samples are not paired. *p* = 7.2 × 10^−4^. Right: TCGA LUAD tumors split by smoking history. FPKM-UQ = Fragments per kilobase of gene per millions mapped, upper quartile normalized. (**C**) Expression of *SULT1C2* in three lung cell lines. RPKM = reads per kilobase of gene per millions mapped. (**D**,**E**) LUAD cell lines were treated with dimethylsulfoxide (DMSO) vehicle control or cigarette smoke condensate (CSC) at 10, 20, 40, and 80 µg/mL. Cells were treated for 24 h, and mRNA expression levels were measured. White = Untreated, gray = DMSO, brown = CSC treatment at indicated doses. (**D**) *SULT1C2* levels in BEAS-2B cells, H2347, and PC3_LUAD cells. (**E**) *CYP1B1* levels in BEAS-2B, H2347, and PC3_LUAD cells. *n* = 3. All qRT-PCR data are normalized to *LACTB* levels in matched cell line. *n* = 3. (*) = *p* ≤ 0.05.

To determine what role *SULT1C2* may play in LUAD, we first utilized large-scale publicly available datasets of gene expression levels in LUAD tumors to determine if *SULT1C2* expression was related to overall patient survival (OS). Because of the known involvement of *SULT1C2* as a xenobiotic metabolism enzyme, we first split patients based on smoke-exposure status, then plotted survival as a function of *SULT1C2* expression using KMplot [47] (Figure 1A). The effect of *SULT1C2* on LUAD patient OS appeared to be dependent on cigarette-smoke exposure. Specifically, *SULT1C2* expression had no effect on patients without smoking history, whereas patients with a smoke exposure history showed improved survival when *SULT1C2* was expressed. To further characterize the relationship between *SULT1C2* and LUAD, we analyzed publicly available data from The Cancer Genome Atlas (TCGA) for LUAD expression in tumor and unmatched adjacent tumor normal (AdjNTL). This indicated that *SULT1C2* expression levels were significantly elevated in LUAD tumors relative to AdjNTL (Figure 1B). To determine the effect of the subjects’ cigarette smoke exposure on *SULT1C2* messenger RNA (mRNA) levels, LUAD tumor samples were split based on patients’ smoke exposure status into four major TCGA-annotated categories: never smokers (category 1), former smokers who quit more than 15 years prior (category 2), former smokers who quit less than 15 years prior (category 3), and current smokers (category 4). Plotting expression of *SULT1C2* relative to smoking status indicated that *SULT1C2* expression levels were significantly inversely correlated to duration of smoke exposure, meaning that the more exposure to cigarette smoke the patient had, the lower expression of *SULT1C2* in their LUAD tumor (*p* = 9.09 × 10^−6^, Figure 1B, Table 2). To investigate the mechanism by which *SULT1C2* expression levels are affected by smoke exposure, we carried out in vitro experiments.

We selected three cell lines with varying levels of *SULT1C2* endogenous expression: immortalized non-cancerous lung epithelial cell line BEAS-2B and two lung adenocarcinoma cell lines, H2347 and PC3_LUAD. PC3_LUAD is a primary lung adenocarcinoma line and has no relation to the ubiquitous prostate cancer cell line PC3. Publicly available RNAseq profiles of all three lung cell lines were downloaded from the Gene Expression Omnibus (GEO) [33,48] and aligned to the hg19 genome prior to determine the levels of reads per kilobase of gene per millions mapped (RPKM). BEAS-2B did not express detectable levels of *SULT1C2* (Figure 1C). H2347 robustly expressed *SULT1C2* while PC3_LUAD expressed barely detectable levels of *SULT1C2* levels as measured by RNAseq (Figure 1C). To determine if cigarette smoke was able to affect transcriptional levels of *SULT1C2,* each of these cell lines was treated with cigarette smoke condensate (CSC). *SULT1C2* RNA levels were measured alongside the positive control gene for CSC exposure, cytochrome p450 1B1 (*CYP1B1*). Untreated cells were maintained in a separate incubator so they would not be affected by secondary aerosolized CSC. All cell lines were treated with 10, 20, 40, and 80 μg/mL of CSC for 24 h. We used 10 and 20 μg/mL to simulate second-hand smoke exposure and 40 and 80 μg/mL to simulate a smoker and heavy smoker environment, respectively, and to account for potential substrate inhibition of the phase I and phase II xenobiotic metabolizing enzymes [49]. We observed a dose-dependent effect on transcription levels of *SULT1C2* in BEAS-2B and H2347 (Figure 1D). In contrast, PC3_LUAD cells showed no significant dose-dependent response. All lung cell lines tested showed a significant transcriptional response of *CYP1B1* at 24 h (Figure 1E). This raised the question of why we observed differential induction of *SULT1C2* in the tested LUAD cell lines and what could account for differential levels of *SULT1C2* expression prior to CSC exposure.

### 3.1. Methylation of SULT1C2 Promoter Is Altered in Human Lung

**Figure 2:**
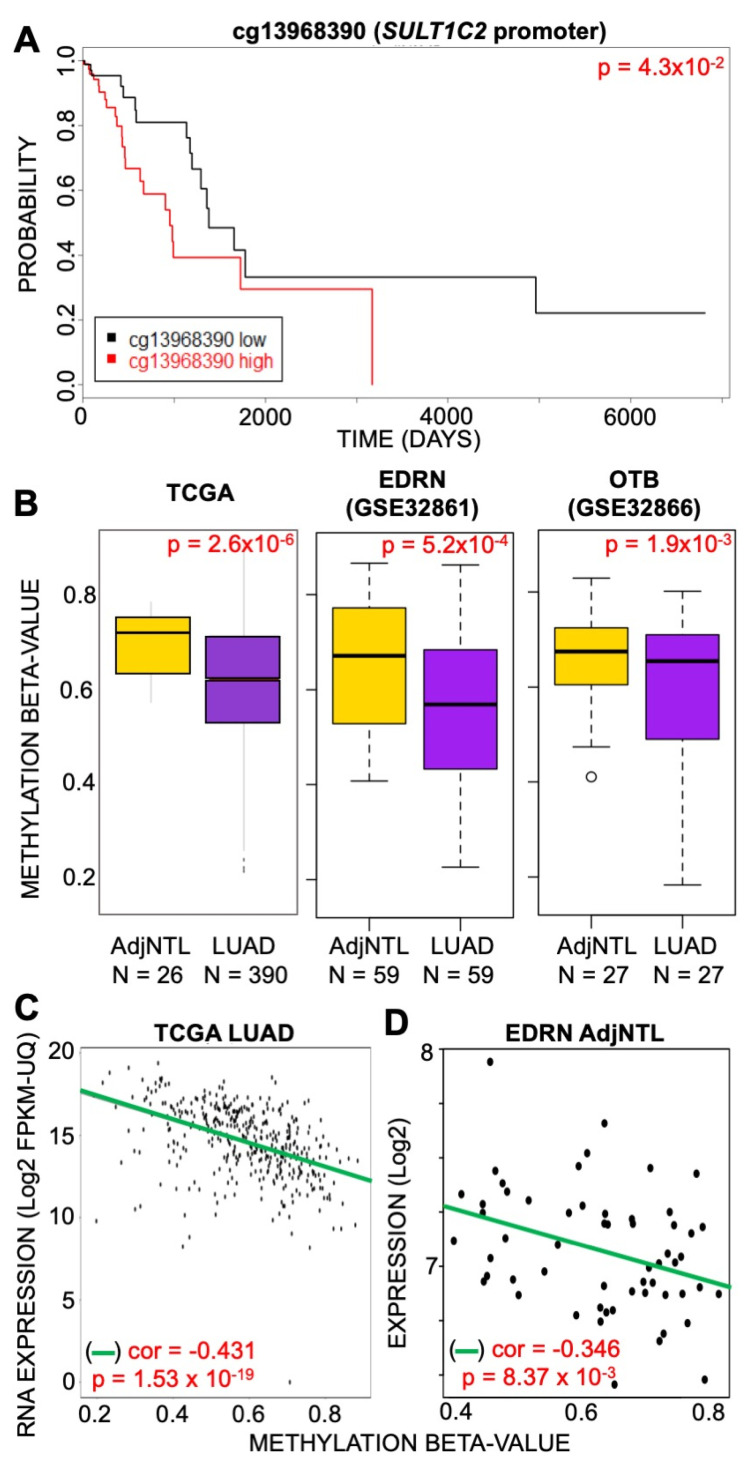
Methylation of *SULT1C2* promoter is altered in human lung. (**A**) Overall patient survival (OS) for samples from the TCGA dataset was split by upper quartile based on methylation at cg13968390 using TCGAbiolinks [43]. (**B**) Boxplots of methylation at cg13968390 derived from TCGA (left), EDRN (middle), and OTB (right) datasets. N = number of samples in collection included in calculations. An unpaired *t*-test was used on TCGA data as the AdjNTL samples are derived from non-matched individuals in that dataset. Paired *t*-tests were used for EDRN and OTB datasets as their AdjNTL was derived from the same patient as the LUAD tumor tissues. Yellow = AdjNTL, purple = LUAD. (**C**) Correlation between expression and methylation in TCGA LUAD tumors (correlation = −0.431, *p* = 1.53 × 30^−19^). (**D**) Correlation between expression (probe ILMN_1772148) and methylation (cg13968390) from the EDRN AdjNTL dataset.

We hypothesized that the differential endogenous expression of *SULT1C2* and response to CSC exposure may be due to differing epigenetic states in the *SULT1C2* promoter-spanning regulatory region. *SULT1C2* is classified as having a CpG-poor promoter, with sparse CpG dinucleotide occurrence. However, multiple studies support a role of CpG-poor promoters in tissue-specific expression [50,51]. To determine the role CpG methylation in the *SULT1C2* promoter plays in LUAD, we first examined DNA methylation profiles of 390 LUAD patients and 26 AdjNTL controls generated by the TCGA on the Illumina Infinium HumanMethylation450 BeadChip [52]. One probe on the array, cg13968390, was located within the *SULT1C2* promoter. Methylation at this probe location was used to split LUAD patients into methylation-high and methylation-low groups, and overall survival (OS) was compared between them. We observed that methylation levels at cg13968390 are significantly inversely correlated with patient OS (Figure 2A). Next, we evaluated if cg13968390 methylation was altered in LUAD tumors compared to AdjNTL. To do so, we again utilized the TCGA LUAD dataset and observed that DNA methylation levels at cg13968390 were significantly lower in LUAD tumors as compared to AdjNTL (two-tailed unpaired *t* test; *p* = 2.6 × 10^−6^, Figure 2B). To validate these findings, we used a secondary, independent dataset generated by the Early Detection Research Network (EDRN) that profiled 59 LUAD tumors alongside matched AdjNTL [44]. We found that cg13968390 was also hypomethylated in LUAD tumors vs. AdjNTL in this dataset (two-tailed paired *t* test, *p* = 5.2 × 10^−4^, Figure 2B). To further validate this finding, we utilized a third, independent study that profiled 27 LUAD tumors and AdjNTL derived from the Ontario Tumor Bank (OTB) tissue repository. We performed a one-sided paired *t*-test to determine if the direction of change in promoter methylation state was similar to the other two datasets and found that cg13968390 was significantly hypomethylated in LUAD tumor tissue from this source as well (*p* = 1.9 × 10^−3^, Figure 2B). Thus, hypomethylation of cg13968390 in tumor compared to non-tumor lung appears to be a common feature of LUAD.

Next, we set out to determine if CpG methylation of the *SULT1C2* promoter was causally related to *SULT1C2* expression. We examined the correlation between expression and methylation of the *SULT1C2* promoter region in samples from the TCGA database for which matched RNA expression and DNA methylation data were available and found a significant inverse correlation between methylation of cg13968390 and *SULT1C2* expression (Figure 2C). Linear regression was used to determine the significance of that association (*p* = 1.53 × 10^−19^). To confirm this observation, a secondary dataset derived from patients in the EDRN collection [53] consisting of 60 tumors and paired adjacent non-tumor lung (AdjNTL) was also examined for methylation state vs. matched *SULT1C2* expression. We again found a statistically significant inverse correlation (cor = −0.346) between cg13968390 DNA methylation and expression of *SULT1C2* (*p* = 8.37 × 10^−6^, Figure 2D). In sum, multiple LUAD patient cohorts exhibited hypomethylation of cg13968390 and a significant inverse relationship to *SULT1C2* expression.

### 3.2. DNA Methylation Represses Transcription of SULT1C2 Promoter

**Figure 3:**
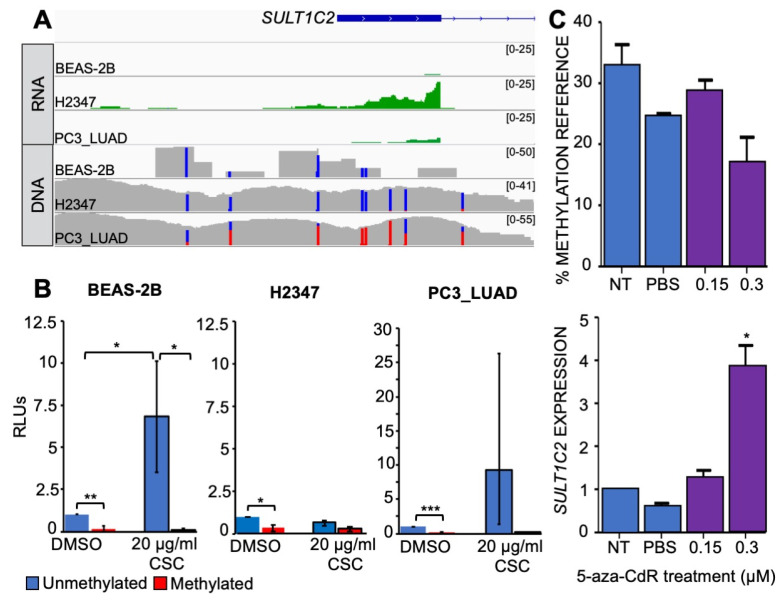
DNA methylation represses transcription of *SULT1C2* promoter. (**A**) IGV browser image of *SULT1C2* promoter region. RNAseq (green) was downloaded from publicly available resources [32,33]. BAM alignment files to hg19 genome displayed. DNA includes RRBS (BEAS-2B) and sWGBS (H2347, PC3_LUAD); blue = unmethylated, red = methylated, gray = non-CpG sequence. (**B**) Cells were transfected with in vitro SssI-methylated or -unmethylated *SULT1C2*-CpGless vector in the indicated cell lines. Cells were subsequently treated with 20 μg/mL CSC for 24 h. Samples were background-subtracted and normalized to CMV-luciferase positive controls as a measure of transfection efficiency. RLUs are expressed as a ratio relative to unmethylated, untreated controls in the indicated cell line. Blue = unmethylated *SULTC2* promoter, red = methylated *SULT1C2* promoter. Black outlines indicate CSC-treated samples. *n* = 3. A two-tailed paired *t* test was used to calculate significance and Bonferroni correction applied for multiple tests; (*) = *p* ≤ 0.05, (**) = *p* < 0.01, (***) *p* < 0.001. (**C**) PC3_LUAD cells, the only cell line in our study with significant endogenous methylation of the *SULT1C2* promoter, were treated with 5-aza-CdR at half the clinical dose (0.15 μM) and the clinical dose (0.30 μM). Pyrosequencing was used to determine differential methylation by percent methylated reference (PMR) to Alu. *SULT1C2* expression was normalized to *LACTB* loading controls. *n* = 3.

Having observed a highly significant correlation between *SULT1C2* promoter hypomethylation in LUAD and *SULT1C2* expression levels, we sought to functionally test this relationship. To do so, we used publicly available RNAseq data for BEAS-2B, H2347, and PC3_LUAD cell lines [33,48] as well as shotgun whole-genome bisulfite sequencing (sWGBS) and reduced-representation bisulfite sequencing (RRBS) data made available on the Database of Transcriptional Start Sites (DBTSS) [38] and the Gene Expression Omnibus (GEO) [36], respectively. Visualization of the *SULT1C2* promoter using bisulfite mode in Integrated Genomics Viewer (IGV) revealed that H2347 cells have unmethylated CpGs throughout the *SULT1C2* promoter and robust expression of *SULT1C2* in untreated cells (Figure 3A) and that PC3_LUAD cells displayed high levels of promoter methylation and low levels of expression, consistent with promoter CpG methylation blocking transcription of the adjacent gene. This may also account for the previously observed inability of CSC to upregulate *SULT1C2* in PC3_LUAD cells (Figure 1E). In contrast, RRBS data generated on BEAS-2B cells indicated that the CpGs present in the RRBS data were unmethylated; however, BEAS-2B cells lack expression of *SULT1C2*. This could be due to a number of factors, such as the requirement for specific transcription factors not expressed in BEAS-2B under untreated conditions or repression of enhancers whose association is required for basal activation of the *SULT1C2* promoter. In order to test whether *SULT1C2* promoter methylation functionally affects transcriptional activity of the adjacent *SULT1C2* gene, the promoter region from −1.27 kb to +535 surrounding the transcriptional start site (TSS) of *SULT1C2* was cloned into a CpG-less vector [54] containing the luciferase reporter gene. The CpG-less vector is devoid of CpG dinucleotides. Therefore, the only CpGs present in the construct are within the *SULT1C2* promoter. The in vitro *SssI*-methylated *SULT1C2* promoter plasmid was transfected into all three cell lines with subsequent CSC treatment to determine the effect of the methylation state of the *SULT1C2* promoter on downstream gene expression levels.

In all three lung cell lines tested, the *SULT1C2* promoter showed baseline activity in the unmethylated state, and this transcriptional activity was repressed by in vitro *SssI* methylation (Figure 3B). Our results are therefore in agreement with Han et al., showing that DNA methylation can directly silence CpG-poor promoters [55]. We then tested whether the addition of CSC could activate the *SULT1C2* promoter. Consistent with the transcript level data in Figure 1, the addition of CSC induced transcriptional activity of the unmethylated *SULT1C2* promoter in BEAS-2B cells. However, we did not observe significant induction by CSC in the two LUAD cell lines, suggesting that while cloned promoter can drive baseline expression, it may lack certain regulatory elements (such as enhancers) mediating CSC induction (Figure 3B).

While the results of the in vitro *SssI*-methylated *SULT1C2* promoter construct indicated that methylation can affect downstream transcriptional activity, this did not test whether alteration of DNA methylation levels in vivo at the endogenous *SULT1C2* locus could alter transcriptional activity of *SULT1C2*. In order to directly test the effect of DNA methylation on the activity of the endogenous promoter, we used the DNA methylation inhibitor 5-Aza-deoxycytidine (5-aza-CdR) to block DNA methylation and subsequently measured *SULT1C2* transcription. PC3_LUAD cells were the only cell line showing endogenous *SULT1C2* promoter methylation in Figure 3A and were therefore the only line used for this experiment, as there was no methylation for 5-aza-CdR to remove in the other cell lines. PC3_LUAD cells were exposed to two doses of 5-aza-CdR, 0.15 µm (half the clinical dose) and 0.3 µm (clinical dose) [56], for 24 h, after which the cells underwent recovery for three days to allow for DNA replication to incorporate the drug into the daughter cells and block DNA methylation [57]. Post treatment with 5-aza-CdR, MethyLight [58] was used to determine the methylation status of the *SULT1C2* promoter alongside qRT-PCR to evaluate *SULT1C2* expression levels. Treatment of PC3_LUAD cells with 5-aza-CdR resulted in a dose-independent decrease in methylation at the endogenous *SULT1C2* promoter (Figure 3C). The percent methylated reference (PMR) decreased and *SULT1C2* gene expression increased in a dose-dependent manner and reached significance at the clinical dose of 0.3 µm. Taken together, these results suggest that methylation plays a significant role in the regulation of *SULT1C2* expression in lung cell lines, which agrees with our findings in human samples that showed an inverse correlation between methylation and RNA expression (Figure 2C).

*SULT1C2* expression is elevated in adjacent non-tumor lung of Asians relative to Caucasians and is significantly correlated to *SULT1C2* promoter methylation levels.

Now that we had established a direct relationship between *SULT1C2* promoter methylation and *SULT1C2* expression, we wanted to further understand what underlying features of the cell models could contribute to the differential promoter methylation observed between PC3_LUAD and H2347. PC3_LUAD and H2347 cell lines were both derived from female patients, and no significant difference was observed in *SULT1C2* levels based on sex or age of the patient (Table 2).

However, expression of *SULT1C2* did vary significantly based on smoking status as expected (Figure 1B). In addition, it varied by the race of the patient in the TCGA cohort, showing increased expression in Asians relative to Caucasians. We then tested the cohort to determine if methylation levels in the *SULT1C2* promoter varied significantly based on race as well. We observed that methylation levels were significantly lower in Asian relative to Caucasian patients (Table 3).

**Figure 4:**
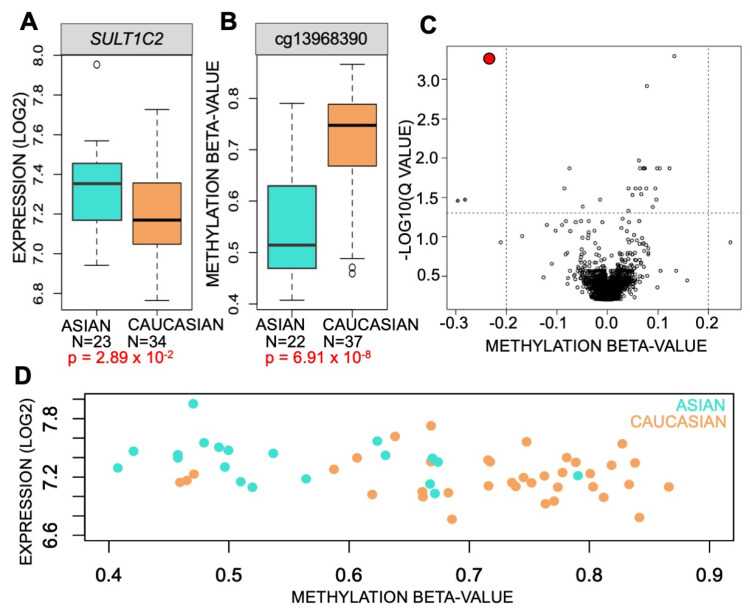
*SULT1C2* expression is elevated in Asian adjacent non-tumor lung relative to Caucasians and is significantly correlated to *SULT1C2* promoter methylation levels. (**A**) Boxplots of *SULT1C2* expression (ILMN_1772148) and (**B**) *SULT1C2* promoter methylation (cg13968390) derived from the EDRN AdjNTL dataset. N = number of samples in collection included in calculations. An unpaired *t*-test was used to calculate significance as the tissues were derived from different patients. Turquoise = Asian, sandy brown = White. (**C**) Genome-wide analysis of significant differences in methylation between Asian and Caucasian in AdjNTL from patients in EDRN. Q values were used for genome-wide FDR correction of significance. Methylation beta-value differences are expressed as changes in Asian methylation levels relative to Caucasian. Red circle = cg13968390. (**D**) Scatterplot of EDRN AdjNTL methylation (cg13968390) versus expression (ILMN_1772148). Turquoise = Asian, sandy brown = White.

However, the TCGA cohort contained relatively few Asian patients, and the DNA methylation data were derived from LUAD which is subject to a host of molecular alterations during tumor formation. To further validate these findings in a secondary dataset with a larger number of Asian patients as well as data from non-tumor adjacent normal tissue, we used the AdjNTL subset of the EDRN cohort, which contained expression and methylation data from adjacent normal lung of 22 Asian and 37 Caucasian patients. We found that *SULT1C2* expression was significantly elevated in Asian relative to Caucasian patients (Figure 4A) and that there was a concomitant lower level of DNA methylation of the *SULT1C2* promoter in Asian patients relative to Caucasians (Figure 4B). Indeed, methylation of cg13968390 within the *SULT1C2* promoter was the most significantly differential methylation event genome-wide between Asians and Caucasians in the EDRN AdjNTL cohort (Figure 4C). To examine the relationship between expression, DNA methylation, and race, we plotted methylation vs. expression of the EDRN dataset and colored each sample by race of the patient, which resulted in two clusters with minimal overlap, one consisting mainly of Asian patients, the other consisting primarily of Caucasian patients (Figure 4D).

### 3.3. CSC Alters AHR Occupancy at the SULT1C2 Promoter

**Figure 5:**
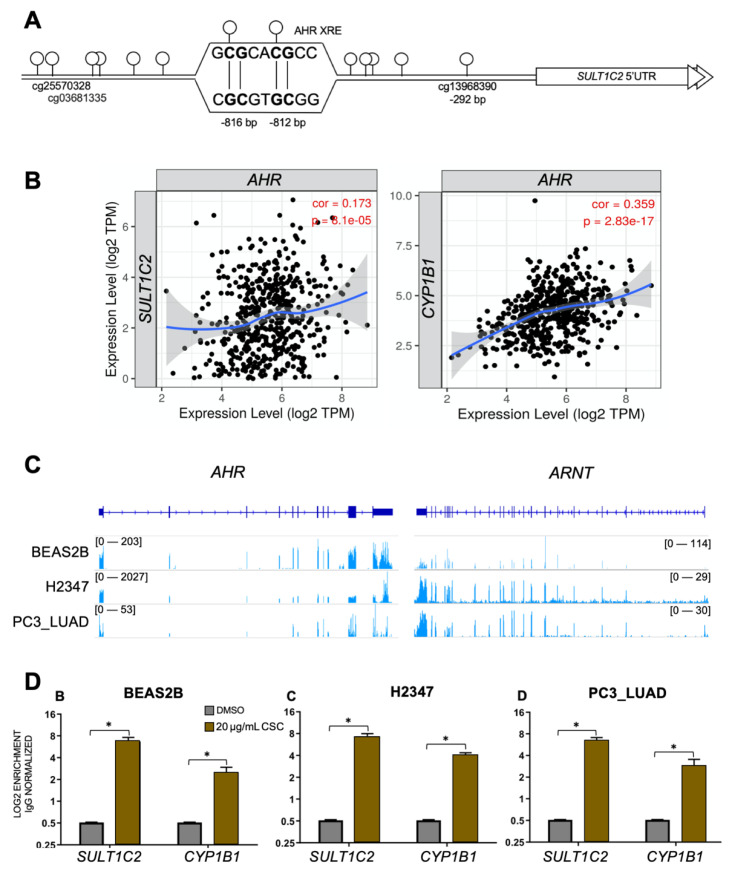
CSC alters AHR occupancy at the *SULT1C2* promoter. (**A**) Diagram of the *SULT1C2* promoter including CpG at cg13968390, including the predicted AHR binding site. Open circles indicate CpG dinucleotides within the 1.8kb promoter region. Coordinates listed are relative to the transcriptional start site of *SULT1C2*. (**B**) Scatterplot of expression for indicated genes. Data included were derived from the TCGA LUAD dataset and visualized using TIMER2.0 [40]. Cor = correlation between expression of the indicated genes. (**C**) IGV browser image of AHR and ARNT genomic regions. RNAseq (blue) was downloaded from publicly available resources [32,33]. (**D**) The indicated cell lines were treated with 20 μg/mL CSC for 72 hrs. ChIP was performed with AHR antibody. Gray = AHR bound to promoter when DMSO treated, Brown = AHR enrichment on *SULT1C2* promoter when treated with 20 µg/mL CSC for 24 h. Normal rabbit IgG was used to correct for background binding levels. (*) = *p* ≤ 0.05.

Now that we had established a direct relationship between *SULT1C2* promoter methylation levels and expression as well as CSC-mediated transcriptional activation, we wanted to understand the mechanism by which CSC mediates activation of the *SULT1C2* promoter. To do so, we performed transcription factor binding site analysis on the area surrounding CpG sites within the *SULT1C2* promoter using Biobase [59] (Figure 5A). We identified the aryl hydrocarbon receptor (AHR) as a likely binding candidate to a site carrying two CpGs within the *SULT1C2* promoter. AHR is a ligand-activated transcription factor [60] whose transcriptional activity is induced by xenobiotic chemicals, among which are polycyclic aromatic hydrocarbons (PAH) such as benzo (a)pyrene found in cigarette smoke [61]. It is well established that aryl hydrocarbon receptor acts as a transcriptional activator for phase I detoxifying enzymes such as *CYP1B1* when induced by exogenous ligands [62], and more recently, it has become accepted that AHR can activate phase II enzymes [19,20,21,22]. We therefore hypothesized that AHR was the major transcription factor bridging CSC-induced transcriptional activity and *SULT1C2* upregulation in cells with unmethylated *SULT1C2* promoters.

If *SULT1C2* is a transcriptional target of AHR in LUAD, we would expect to see a positive correlation between *AHR* and *SULT1C2* expression in LUAD patient data cohorts. To test this, we utilized RNA expression levels of *AHR* and *SULT1C2* generated by the TCGA PanCancer study through the Tumor-Immune Estimation Resource (TIMER2.0) portal [40]. Indeed, *SULT1C2* expression levels were significantly positively correlated to *AHR* expression in LUAD (cor = 0.173, *p* = 8.1 × 10^−5^, Figure 5B). This trend was also observed in the known AHR target gene *CYP1B1* (cor = 0.359, *p* = 2.83 × 30^−17^).

We then sought to test if AHR bound differentially to the *SULT1C2* promoter in the presence of cigarette smoke. In order to do so, we first analyzed RNAseq from BEAS-2B, H2347, and PC3_LUAD for levels of *AHR* expression. Indeed, all three cell lines expressed *AHR* and its dimerization partner, *ARNT* (Figure 5C). We then performed chromatin immunoprecipitation of AHR from cell lines treated with vehicle or 20 μg/mL CSC. CSC treatment resulted in significant enrichment of AHR at the *SULT1C2* promoter in all three cell lines as compared to DMSO (Figure 5D). In all cases, enrichment of AHR binding to the *SULT1C2* promoter was greater than the enrichment at a previously described AHR binding site near *CYP1B1* [62].

## 4. Discussion

*SULT1C2* is a phase II detoxifying enzyme known for its ability to metabolize xenobiotics by adding a sulfonate group to the target, facilitating excretion via urine or bile. In this study, we made several observations. First, that CSC was able to induce *SULT1C2* expression in lung cells and that this activation was cell-line dependent. Sakakibara et al. previously observed *SULT1C2* in fetal lung tissue [63]; however, there is little to no expression of *SULT1C2* in the human adult lung as reported by multiple consortia, including the Human Protein Atlas [64], GTEx [65], and FAMTOM5 [66] projects. We detected expression of *SULT1C2* in the AdjNTL of Asian patients and in one of the LUAD cell lines, H2347, without exposure to CSC. This may be due to carcinogenic processes affecting the AdjNTL samples, as they exist in proximity to the tumor, or to environmental toxins involved in the etiology of the individuals’ LUAD development. The presence of *SULT1C2* in these normal lung samples would predispose affected individuals for conversion of substrates into metabolically activated electrophiles that have the potential to be both carcinogenic and mutagenic [12,13]. The amount of cigarette smoke inhaled could also be of great significance. Phase II enzymes can become over-saturated with substrate and experience substrate inhibition. In our cell-line study, we used several levels of CSC to simulate various smoking conditions and found that very low levels, mimicking exposure of second-hand smokers, had the greatest increase in expression of *SULT1C2*.

Secondly, we also determined that methylation of the CpG-poor *SULT1C2* promoter regulated downstream transcriptional activity. The paradigm surrounding the function of DNA methylation at CpG-poor promoters is now shifting toward the idea that both CpG-poor and CpG-rich promoters appear to be repressed by DNA methylation [51,55]. We further observed that CSC can stimulate *SULT1C2* promoter activity and that this stimulation was lost when the promoter was methylated in vitro. It is well known that cigarette smoke can alter DNA methylation [67,68,69]. We now demonstrate that DNA methylation can affect the cigarette smoke response; it can inhibit the ability of CSC to activate gene expression of a key member in the detoxification pathway.

Third, we discovered that race can play a significant role in levels of DNA methylation at the *SULT1C2* promoter as well as overall expression of the *SULT1C2* gene in non-tumor lung tissue. We observed hypomethylation of the *SULT1C2* promoter in AdjNTL from Asian relative to Caucasian patients, with a correlative increase in expression, in data from both TCGA and EDRN consortia. This could indicate a unique underlying predisposition for people of Asian descent to have hypomethylated and expressed *SULT1C2* in their lung tissue without the activating event of environmental toxin exposure. This could contribute to the well-documented racial disparities in clinical presentation of LUAD in these different patient populations.

Lastly, we determined that the transcription factor AHR has a DNA binding site in the promoter of *SULT1C2* and binding of AHR to the *SULT1C2* promoter is activated by cigarette smoke. Indeed, the fold enrichment of AHR on the *SULT1C2* promoter was significantly greater than that of the previously characterized AHR binding site at *CYP1B1* [62]. While we established that AHR was expressed in all three cell lines prior to CSC treatment, it is possible that total AHR levels could also be upregulated in response to CSC exposure. Indeed, AHR levels have been previously reported to change in response to cellular xenobiotic exposure [20]. Future studies could characterize three-dimensional interactions between transcription factor complexes assembled on the *SULT1C2* promoter to examine any possible interactions between DNA methylation regulatory complexes and AHR DNA binding sites that interact to coordinate cellular responses to xenobiotic compounds by activating sulfotransferases.

Our study was confined to characterizing the regulatory mechanisms at play on the *SULT1C2* promoter in lung tissue. One of the limitations of our study was that we did not characterize *SULT1C2* enzymatic activity in lung, nor did we determine if *SULT1C2* was able to metabolize in the lung the same way that has been previously characterized in the liver. Studies will be needed to determine the effects of baseline *SULT1C2* levels on the metabolism of CSC and other carcinogens and downstream tumorigenesis. In addition, our study did not explore the mechanistic implications of AHR binding to the *SULT1C2* promoter and if this is affected by a wide range of xenobiotic chemicals beyond CSC. General trends in tobacco usage have been shifting in the United States for some years, and it remains unclear if e-cigarettes or other inhaled compounds can elicit the same activation of AHR and upregulation of *SULT1C2*. Usage of specific types of cigarettes, e-cigarettes, and other inhaled compounds is documented to vary by race and therefore could influence the observed effect of race on *SULT1C2* transcriptional activity. Our study did establish that there is a difference in the overall methylation of *SULT1C2* promoter and that this methylation difference affects the ability of CSC-activated AHR to bind and activate *SULT1C2* expression. Further studies to determine the underlying drivers of this methylation difference may yield key answers in our understanding of how epigenetic differences between races can affect disease outcomes.

## 5. Conclusions

In this article, we analyzed *SULT1C2* expression in LUAD and lung cell lines. Bioinformatic analysis showed that *SULT1C2* expression in LUAD is correlated to improved patient survival but only in patients with cigarette smoke exposure, while corresponding hypermethylation of the *SULT1C2* promoter was correlated with poorer overall patient survival. DNA methylation was able to inactivate downstream *SULT1C2* transcription. In addition, we found that the normal lungs of Asian patients have elevated levels of *SULT1C2* and lower methylation of the *SULT1C2* promoter. CSC-activated AHR was bound to the *SULT1C2* promoter specifically in the unmethylated state, which in turn activated downstream transcription of *SULT1C2*. Taken together, our results suggest a mechanism by which methylation of the *SULT1C2* promoter and subsequent disruption of binding of the aryl hydrocarbon receptor disrupts transcriptional activation of the *SULT1C2* detoxification enzyme and thereby affects the cell’s ability to respond to cigarette smoke exposure.

Our analyses indicate a complex role for *SULT1C2* in lung adenocarcinoma. On the one hand, higher *SULT1C2* expression in LUAD is associated with improved survival, which would be in line with the detoxification role of the encoded enzyme. On the other hand, we detected higher expression of *SULT1C2* in the AdjNTL of Asian vs. Caucasian patients. Because *SULT1C2* can also activate the carcinogenic potential of xenobiotics, this may point to a potential role in the etiology of LUAD in these patients, who are largely non-smokers, but who could be exposed to second-hand smoke or other environmental agents. Taken together, our data indicate that the interplay of DNA methylation, tobacco smoke exposure, and *SULT1C2* expression could play a pivotal role in the etiology and racial disparities observed in LUAD.

## Figures and Tables

**Table 1:** Primers.

*LACTB* Forward	5′-GTTGAGAACCGTGTACCATGT-3′
*LACTB* Reverse	5′-TTCCCACAATTTGGCAAGAGC-3′
*SULT1C2* Forward	5′-CAGCCTGCAACTGTGGACAA-3′
*SULT1C2* Reverse	5′-GATGGCGGTGTTGGATGATG-3′
*CYP1B1 Forward*	5′-CTGCACTCGAGTCTGCACAT-3′
*CYP1B1 Reverse*	5′-TATCACTGACATCTTCGGCG-3′
*SULT1C2* promoter Forward	5′-aaaaaaactagtCATCCCAGTTCATCCTCCACAAA-3′
*SULT1C2* promoter Reverse	5′-aaaaaatcatgaTTTGAATAAATGCATCTGTAAAGCCA-3′
MethyLight *SULT1C2* Forward	5′-GGGTATGGTGGCGTACGTT-3′
MethyLight *SULT1C2* Reverse	5′-AATCTTAACTCACTACAACCTCCG-3′
MethyLight *SULT1C2* Probe	5′-/6FAM-CTCCCGAATTCAAACGATTCTCCTATCTCA-BHQ-3/-3
MethyLight *ALU* Forward	5′-AGGTCGAGGTCGGCGG-3′
MethyLight *ALU* Reverse	5′-CCACGCCCGACTAATTTTATATCTT-3′
MethyLight *ALU* Probe	5′-/6FAM-CAAACTAATCTCAAACTCCCGACCTCAAACGA-BHQ-1/-3′
ChIP *SULT1C2* Forward	5′-CCGTCTCTACTAAAAATACGAA-3′
ChIP *SULT1C2* Reverse	5′-AGCGATTCTCCTGTCTCAGCC-3′
ChIP *CYP1B1* Forward	5′-ATATGACTGGACCGACTTTCC-3′
ChIP *CYP1B1* Reverse	5′-GGCGAACTTTATCGGGTTGA-3′

**Table 2:** *SULT1C2* expression in TCGA LUAD. Multiple linear regression was used to include all listed clinical features into one model. Bolded *p* values were considered significant.

*SULT1C2 Expression*	Number of Patients	Estimate	*p*-Value
Sample type			
*Normal*	53	0.60538	0.0479
*Tumor*	429		
Gender			
*Male*	210	0.13455	0.4926
*Female*	272		
Age		*By Year*	
	482	0.01027	0.3031
Smoking		vs. *Never Smoker*	
*Never Smoker*	71	--	--
*Former Smoker (≥15 years)*	129	−0.05579	0.8595
*Former Smoker (<15 years)*	169	−0.70430	**0.0187**
*Current Smoker*	113	−1.03470	**0.0016**
Race		vs. *Caucasian*	
*Caucasian*	422	--	--
*Black or African American*	52	0.39562	0.2068
*Asian*	7	1.63644	**0.0414**
*American Indian or Alaskan Native*	1	−0.12833	0.9512

**Table 3:** The effect of race on *SULT1C2* promoter methylation levels in TCGA LUAD dataset. Univariate linear regression was used on the indicated clinical feature. Bolded *p* value was considered significant.

*cg13968390 Methylation*	Number of Patients	Estimate	*p*-Value
Race		vs. *Caucasian*	
*Caucasian*	359	--	--
*Black or African American*	51	0.005096	0.8020
*Asian*	6	−0.130799	0.0197
*American Indian or Alaskan Native*	0	N/A	N/A

## Data Availability

All next-generation sequencing and microarray data used in this manuscript are publicly available. Microarray data on LUAD expression and methylation generated by the Early Detection Research Network (EDRN) and Ontario Tumor Bank are available at GEO as parts of SuperSeries GSE32867 [44] (https://www.ncbi.nlm.nih.gov/geo/query/acc.cgi?acc=GSE32867 accessed on 15 July 2021). Shotgun whole-genome bisulfite sequencing (sWGBS) on PC3_LUAD and H2347 cells is available for download from the DataBank of Transcriptional Start Sites (DBTSS) website (https://dbtss.hgc.jp/ accessed on 15 July 2021) [38]. Enhanced reduced-representation bisulfite sequencing (eRRBS) on BEAS-2B is available from GEO (GSE155615) [36] (https://www.ncbi.nlm.nih.gov/geo/query/acc.cgi?acc=GSE155615 accessed on 15 July 2021). Data from The Cancer Genome Atlas on LUAD expression, methylation, and smoking status are available through the Genomics Data Portal (https://portal.gdc.cancer.gov/ accessed on 15 July 2021) and accessible via the TCGAbiolinks package in R [43]. Bulk RNAseq is available at GEO for BEAS-2B (GSE55215) [48] (https://www.ncbi.nlm.nih.gov/geo/query/acc.cgi?acc=GSE55215 accessed on 15 July 2021) as well as H2347 and PC3_LUAD (GSE110024) [33] (https://www.ncbi.nlm.nih.gov/geo/query/acc.cgi?acc=GSE110024 accessed on 15 July 2021).

## Abbreviations

CSC	cigarette smoke condensate
LUAD	lung adenocarcinoma
AdjNTL	adjacent non-tumor lung
XRE	xenobiotic response element
AHR	aryl hydrocarbon receptor
PAH	polycyclic aromatic hydrocarbons
5-aza-CdR	5-Aza-deoxycytidine
ETS	environmental tobacco smoke
TSS	transcriptional start site
ALU	arthrobacter luteus repetitive element
RLU	relative light units
